# Identification of Signal Bias in the Variable Flip Angle Method by Linear Display of the Algebraic Ernst Equation

**DOI:** 10.1002/mrm.22849

**Published:** 2011-03-22

**Authors:** Gunther Helms, Henning Dathe, Nikolaus Weiskopf, Peter Dechent

**Affiliations:** 1 MR-Research in Neurology and Psychiatry, University Medical CentreGöttingen, Germany; 2 Biomechanics Group, Department of Orthodontics, University Medical CentreGöttingen, Germany; 3 Wellcome Trust Centre for Neuroimaging, UCL Institute of Neurology, University CollegeLondon, United Kingdom

**Keywords:** *T*_1_-relaxation, quantification, Ernst equation, variable flip angle experiment

## Abstract

A novel linear parameterization for the variable flip angle method for longitudinal relaxation time *T*_1_ quantification from spoiled steady state MRI is derived from the half angle tangent transform, τ, of the flip angle. Plotting the signal *S* at coordinates *x* = *Sτ* and *y* = *S*/τ, respectively, establishes a line that renders signal amplitude and relaxation term separately as *y*-intercept and slope. This representation allows for estimation of the respective parameter from the experimental data. A comprehensive analysis of noise propagation is performed. Numerical results for efficient optimization of longitudinal relaxation time and proton density mapping experiments are derived. Appropriate scaling allows for a linear presentation of data that are acquired at different short pulse repetition times, TR << T1 thus increasing flexibility in the data acquisition by removing the limitation of a single pulse repetition time. Signal bias, like due to slice-selective excitation or imperfect spoiling, can be readily identified by systematic deviations from the linear plot. The method is illustrated and validated by 3T experiments on phantoms and human brain. Magn Reson Med, 2011. © 2011 Wiley-Liss, Inc.

The degree of longitudinal relaxation time (*T*_1_) weighting in spoiled gradient echo MRI (1) is imposed by the partially saturated steady state that is determined by the interaction of flip angle and *T*_1_ relaxation during the repetition time, TR, (2,3). This leads to the well-known transition from proton density weighting to *T*_1_ weighting. The flip angle dependence is exploited in the most common quantitative MRI application that estimates *T*_1_ by variable flip angles, VFA, (4–8) or its dual flip angle version. After scaling by the sine and the tangent of the flip angle a linear relationship between the corresponding signals is established (5) allowing for computationally efficient linear programing. Recent research on the VFA method focused on noise optimization (9–12) and especially bias reduction (13–15).

We have recently proposed an exact algebraic formulation of the Ernst equation for the spoiled steady state signal (16). A simple rational function is obtained after suitable nonlinear transformations in flip angle and TR/*T*_1_. For small flip angles or short TR, these transformations conform to proportionality with a third order error. Thus, the algebraic signal equation is particularly useful for fast low-angle shot (FLASH) applications (17). Here, we show that the algebraic Ernst equation provides a highly conspicuous linear display of VFA data. Intercept and slope represent signal amplitude and relaxation term, respectively. Thus, deviating signals, due to residual transverse coherences, can be easily identified by visual inspection. This article gives a full account of the underlying theory and experimental validation by measurements at 3 Tesla.

## Theory

The common Ernst equation applies to the situation where the transverse coherences are irreversibly dephased, that is, perfect “spoiling.” For given flip angle, α, and TR, the signal is given by


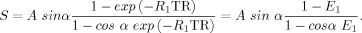
[1]

Here and in the following, we use the relaxation rate*, R*_1_ = 1/*T*_1_, and *E*_1_ = *exp*(−*R*_1_TR) to simplify the notation. *A* = *A*(TE) denotes the amplitude of the gradient echo at the echo time, TE, after excitation by α = π/2 under fully relaxed conditions (TR ≫ *T*_1_). As first suggested in (5), the Ernst Eq. 1 can be rearranged into


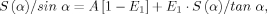
[2]

to derive *R*_1_ and A from the linear relationship between *S*/sin α and *S*/tan α.

We recently employed the half-angle tangent substitution of the trigonometric functions for arbitrary flip angles below π (16)


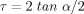
[3]

and an analogous hyperbolic tangent substitution of the relaxation term


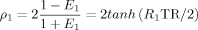
[4]

to transform the Ernst Eq. 1 into a low order rational function in τ and ρ_1_:


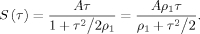
[5]

**FIG. 1:**
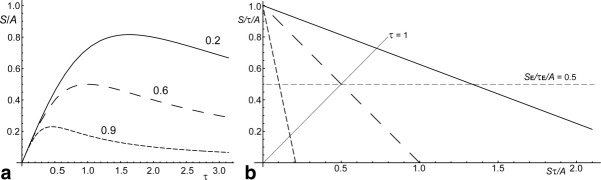
The algebraic signal equation and its linear plot. **a**: The normalized signal plotted for three values of *exp*(–*R*_1_TR): 0.9 (short dashes, *R*_1_TR = 0.10, ρ_1_ = 0.105); 0.6 (long dashes, *R*_1_TR = 0.511, ρ_1_ = 0.5); and 0.2 (solid, *R*_1_TR = 1.61, ρ_1_ = 1.33). Note that α = π is projected to infinity. **b**: The same examples as in **a** are shown after linear parameterization. The *y*-intercept corresponds to *S*/τ = *A* toward α = 0. The *x*-intercepts correspond to the data toward α = π. Ernst conditions are met at *S*/τ/*A* = 0.5 (dashed horizontal line). The line through the origin represents the signals measured at τ = 1. As seen in **a**, these fall beyond the Ernst angle (short dashes), right onto it (long dashes), and below it (solid line).

The symbol ρ_1_ must not be confused with the proton density, which is inherent to the signal amplitude. This function is shown in Fig. 1a. By the transformations [3] and [4], the range of sequence parameters is transformed from (0 < α < π) and (0 < *R*_1_TR < ∞) to (0 < τ < ∞) and (0 < ρ_1_ < 2). Toward zero, the transformations [3] and [4] conform to α and *R*_1_TR, respectively, with a third order error. The Ernst angle in terms of τ is determined by the root of the derivative with respect to τ:


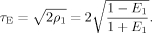
[6]

This yields the maximum signal


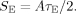
[7]

Since Eq. 5 can be normalized with respect to *S*_E_ and τ_E_, it follows that


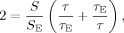
[8]

showing that there is a linear relationship between *S*(τ)τ and *S*(τ)/τ. After division by τ and multiplication of the denominator, Eq. 5 can be rearranged as


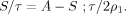
[9]

The convenient features of this linear plot of the ordinate *y* = *S*(τ)/τ against the abscissa *x* = *S*(τ)τ are illustrated in Fig. 1b: The negative slope depends only on the relaxation term ρ_1_. The intercept of the ordinate is identical to the signal amplitude, being the limit of *S*(τ)/τ for τ→0. The intercept of the abscissa corresponds to an infinite τ or α = π. The maximum signal at the Ernst angle is always found halfway between the intercepts since Eq. 7 can be rewritten as *y* = *S*_E_/τ_E_ = *A*/2.

Any fractional signal level *p*, given by pA = *S*(τ)/τ = *pA*, corresponds to a multiple of τ_E_:


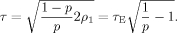
[10]

If *N* measurements are to be performed to yield *N* equidistantly spaced *p*(*i*), excluding the values 0 and 1, *p*(*i*) are given by *i*/(*N* + 1). For these, Eq. 10 yields


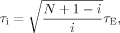
[11]

which in turn can be converted into the corresponding flip angles. Compromises can be imposed on equidistantly sampling the actual signal behavior by *N* measurements, if the MR system restricts the choice of flip angles to integer values. Note that because τ_*i*_/τ_E_ = (τ_N+1−*i*_/τ_E_)^-1^, the corresponding signals should be equal.

For *N* signals *S*_*i*_ at arbitrary flip angles α_*i*_, the relaxation parameter ρ_1_ is calculated from the regression formula of the slope of *y* over *x.*


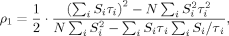
[12]

with τ_*i*_ obtained from α_*i*_ by Eq. 3. This is then transformed into


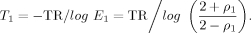
[13]

The amplitude is given by the standard formula of the *y*-intercept:


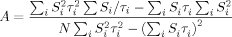
[14]

The case *N* = 2 yields the dual flip angle solutions presented in (16).

### Propagation of Signal Noise

For any given set of independently measured signals *S*_*i*_ the propagation of signal noise (σ_S_) into the ρ_1_ maps can be directly from calculated Eq. 12 as


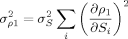
[15]

and correspondingly for the *A* maps from Eq. 14. This determines the signal-to-noise ratio (SNR) of the parameter maps. For *N* ≥ 3, the polynomial order is too high to be solved analytically.

Since the algebraic formulation is computationally efficient, the optimal settings for τ/τ_E_ and the corresponding minima in the normalized variances (Table 1) were obtained by gradient search for a grid of starting values programed in Mathematica 4.0 (Wolfram Research Europe, Long Hanborough, Oxon, UK). To estimate ρ_1,_ an even *N* required a repetition of the optimized dual angle experiment with the variance proportional to 1/*N*. Schemes with uneven *N* were less signal-to-noise efficient, but also required only two flip angles, below and above the Ernst angle. For *A*, a similar dichotomy was observed; however, the errors decreased with the number of points measured below the Ernst angle. Thus, *N* – 1 low flip angles yielded the absolute noise minimum, which decreased stronger than 1/*N*.

**Table 1:** Numerically Derived Settings for Minimal Noise Propagation

*N*	σ^2^_ρ1_ × *A*^2^/ρ_1_^2^/ σ^2^_S_^a^	τ_1opt_/τ_E_	τ_2opt_/τ_E_	σ^2^_A_ × ρ_1_^2^/ σ^2^_S_^a^	τ_1Aopt_/τ_E_	τ_2Aopt_/τ_E_
2	**4.000**	0.4142	2.4142	**5.6133**	0.4903	3.1461
3	2.9313	0.3718	2.2108	2.9886	0.4633	2.7927
		0.3718			0.4633	
3	2.9313	0.4520	2.6939			
			2.6931			
4	**2.0000**	0.4142	2.4141	**2.8067**	0.4904	3.1721
		0.4142	2.4141		0.4903	3.1495
4				2.0841	0.4456	2.6310
					0.4456	
					0.4456	
5	1.6532	0.3898	2.2885	1.6203	0.4323	2.5303
		0.3898	2.2892		0.4323	
		0.3898			0.4323	
					0.4323	
5	1.6532	0.4370	2.5664			
		0.4371	2.5656			
			2.5633			
6	**1.3333**	0.4142	2.4142	**1.8711**	0.4903	3.1483
		0.4142	2.4142		0.4905	3.1471
		0.4142	2.4142		0.4904	3.1471
6				1.3360	0.4217	2.4590
					0.4217	
					0.4217	
					0.42165	
					0.42165	

a Normalized unit-free variances and normalized τ as defined in (16). The left side of the look-up table pertains to noise propagation into the normalized maps of ρ_1_ (relaxation), the right side to normalized maps of *A* (amplitude).

Variation in the last digits is due to the cut-off condition of the algorithm and rounding.

Note that the unit-free normalized variances for the symmetric schemes (boldface) are proportional 1/*N*, because the dual angle settings are replicated.

If *T*_1_ is to be mapped with a certain number of measurements for a given TR and a target value of *T*_1_, ρ_1_ and τ_E_ should be calculated from Eqs. 4 and 6, so the recommended values of the single τ_i_ can be obtained from the table and the respective values of the flip angles are obtained from Eq. 3.

### Noise Propagation with Correlated Errors

The image noise is scaled individually along abscissa (σ_S_τ) and ordinate (σ_S_/τ). The errors are equal for τ = 1 or α = 53°. At smaller flip angle (τ → 0), the *y*-error is enhanced and the *x*-error is diminished, and vice versa for τ → ∞ (α → π).

In a function *F* of two variables, *x* and *y*, the statistical errors (given by the variances *var x* and *var y*) are propagated by the respective partial derivatives ∂*F*/∂*x*. If the errors in *x* and *y* are not independent (i.e., correlated), their covariance has to be taken into account by using the general law of Gaussian error propagation.


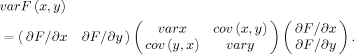
[16]

Here, *F* is the residual *F*(*x*,*y*) = *y* – (*A* – *x*/2ρ_1_) with *x* = *S* × τ and *y* = *S*/τ. Thus, the covariance is identical to the image noise variance σ_S_^2^ and independent of τ.


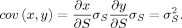
[17]

This is in stark contrast to the common linearization of Eq. 2, where the errors and their covariance increase strongly for small flip angles by 1/sin α or 1/tan α (10). Equation 16 yields


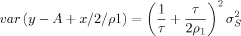
[18]

The expression in brackets denotes a τ-dependent scaling factor of the image noise. It can be cancelled by imposing suitable weights *w*(τ) onto the square residues


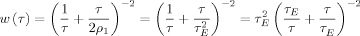
[19]

in the linear least squares objective function. These weights are maximal at τ_E_ and decrease towards the intercepts with the *x*- and *y*- axes.

### Short TR

If *R*_1_TR ≪ 1 then ρ_1_ can be approximated and Eq. 9 arranged as


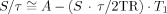
[20]

Under this condition, *Sτ*/2TR can be used as abscissa to incorporate varying values of TR into the regression. Estimates of *T*_1_ are obtained directly as the (negative) slope


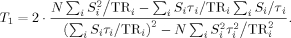
[21]

This is the generalization of the formula for the dual angle experiment (18).

### Flip Angle Inhomogeneities

If the size of the imaged object is of the order of the radio-frequency (RF) wavelength, the transmitted RF field and thus the flip angle is not homogeneous across the object. The (local) signal in 3D MRI is then determined by the spatial distribution of the actual flip angle


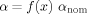
[22]

The factor *f*(x) denotes the bias field describing miscalibration, flip angle inhomogeneity, or the profile of slab-selective excitation. It has to be determined by suitable techniques (19) and taken into account when calculating τ_*i*_. Otherwise, it will give rise to systematic errors in ρ_1_ and *A*. In good approximation, τ can be replaced by α (in radian) in Eqs. 9 and 12 for small flip angles as usually used in FLASH (16,17). At 40° flip angle, τ is underestimated by 4%. Apparent estimates of ρ_1_ and the corresponding relaxation time.


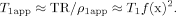
[23]

are then calculated from the nominal flip angles in Eqs. 12 or 21. It can be corrected post hoc by division of the flip angle bias field *f*(*x*)^2^ (17) or by applying a unified segmentation algorithm (20) for bias correction (21).

In slice-selective 2D excitation, the signal represents the distribution of flip angles across the slice profile, which is difficult to describe analytically. This also applies to the residual unspoiled transverse magnetization. For such problems, appropriate phenomenological correction methods of *T*_1_ have been suggested (14,22).

## MATERIALS AND METHODS

Experiments were performed on a 3T clinical MR system (Magnetom Tim Trio, Siemens Healthcare, Erlangen, Germany). A transmit-receive knee coil was used for phantom measurements, and an eight-channel receive-only head coil (Invivo, Gainesville, FL) with body coil transmission for human studies. Three-dimensional FLASH MRI with nonselective excitation was performed to avoid additional bias related to the slice profile (see below). This customized sequence (21) allowed control of the phase increment used for RF spoiling. One healthy adult subject was examined after giving informed consent as approved by the ethics committee of the Göttingen University Medical Center.

### Experiment 1: Nonselective Excitation

To illustrate nearly ideal behavior, experiments were performed on 6% agar (by weight, Sigma-Aldrich Sverige AB, Stockholm, Sweden) in a spherical phantom of 10 cm diameter. *T*_1_ = of 2.41 ± 0.36 sec and *T*_2_ = 20 ± 1 ms were determined on two different occasions by single-slice 5-point inversion recovery and single-volume localization by a stimulated echo at logarithmically increasing TE = 20, 30, 45, 65, 95, 135 ms as described in (23). The agar reduces bulk motion and the *T*_2_/*T*_1_ ratio, thus reducing the effect of residual transverse coherences of echo signals when compared with brain tissue. The agar phantom was covered by a 128 × 104 × 104 matrix with 1 mm isotropic resolution. The nominal flip angle of the vendor's product FLASH sequence (“gre”) was varied between 1° and 34°. The flip angle range was adjusted according to each of the four different values of TR (6, 12, 24, and 48 ms) to avoid crowding (6 ms: 1°–13°; 12 ms: 1°–18°; 24 ms: 1°–24°; 48 ms: 2°–34°). Use of a nonselective rectangular RF pulse and a read-out bandwidth (BW) of 270 Hz/pixel resulted in a TE of 2.65 ms.

### Experiment 2: Slice-Selective Excitation

To study the effect of slice-selective excitation in the vendor's FLASH sequence, bandwidth, TE, and TR were increased to 435 Hz, 3.2 ms, and 7 ms, respectively, when compared with Exp. 1. Central slices of 5 mm thickness were imaged on the agar phantom using the excitation pulse shapes (“normal” and “fast”) as implemented in the vendor's “gre” FLASH sequence and compared to 3D encoding of 52 partitions across a slab of 104 mm, that is, 2 mm partition thickness.

### Experiment 3: RF Spoiling at Varying Phase Increment

The effect of RF spoiling was studied on a 0.1 mM solution of MnCl_2_ to smooth out the rapid variation in the dependence of *T*_1_ on the phase increment (15). *T*_1_ = 0.54 ± 0.37 s and *T*_2_ = 39.7 ± 0.2 ms were determined on two different occasions by single-slice 6-point inversion recovery and single-volume localization by a stimulated echo at logarithmically increasing TE = 20, 30, 45, 65, 95, 135, 200, 300 ms. In contrast to Exps. 1 and 2, a customized 3D FLASH sequence implemented in IDEA VB15, Siemens Healthcare, (21,24) was used for acquisition at TR = 7 ms, TE = 2.6 ms, bandwidth = 305 Hz/pixel. Based on the extensive simulations in (14), we chose to study two intervals of different qualitative behavior: First, the phase increment was varied from 117.0° to 119.8° in steps of 0.4° to sample the rapid transition from negative to large positive residuals (i.e., deviations from the Ernst equation assuming perfect spoiling). Then, the less variable range between 47° and 54° phase increment was sampled in steps of 1°. This experiment comprises the 50° default increment of the “gre” sequence. For each phase increment, measurements were performed at VFA of 2°, 3°, 4°, 6°, 8°, 10°, 12°, 15°, 18°, 21°, 24°, 30°, and 36°.

### Experiment 4: Human Brain In Vivo

In vivo, an isotropic resolution of 1.25 mm and 6/8 partial Fourier acquisition was chosen to reduce the measurement time. 128 sagittal partitions of 192 × 174 pixels were acquired at a constant TR of 11 ms in 3:10 minutes per volume. The TE of 4.92 ms at a bandwidth of 200 Hz/pixel yielded fat and water signals in phase. The phase increment was 50°. These settings have been used in a scheme to correct magnetization transfer images for the influence of *T*_1_ and RF inhomogeneities (25). The flip angle was varied through a total of 16 values from 2° to 12° to 24° to 40° to 60° in steps of 2°, 3°, 4°, and 10°, respectively. A *T*_1_-weighted MP-RAGE scan (magnetization-prepared rapid acquisition of gradient echoes; TI = 900 ms, α = 9°, TE = 3.2 ms, TR = 2250 ms) of 1 mm isotropic resolution was acquired as an individual anatomical reference. Rapid flip angle mapping was performed as described previously (26).

### Data Processing

Mean signal and the standard deviation were assessed over a 10 mm region-of-interest in the center of the phantoms. Kaleidagraph 3.6 for Windows (Synergy Software, Reading, MA) was used for spreadsheet calculations, display, and least-squares fitting with optional weighting of the squared residuals. For nonlinear fitting, a modified Levenberg-Marquardt algorithm with numerical estimation of partial derivatives yielded the Cramér-Rão lower bound estimates for the standard errors.

In vivo data were processed using the FMRIB software library (FSL 4.1, Center for Functional Magnetic Resonance Imaging of the Brain, University of Oxford, UK http://www.fmrib.ox.ac.uk/fsl). The MP-RAGE volume was aligned to the 1 mm brain template of the Montreal Neurological Institute by a rigid-body transform. The VFA data were then aligned to this individual reference. 3D region-of-interests were placed in the head of the left caudate nucleus, the splenium of the corpus callosum, and the left lateral ventricle to evaluate signals of gray matter (GM), white matter (WM), and cerebro-spinal fluid (CSF), respectively. Maps of *T*_1_ were obtained by linear regression of signals that were unaffected by signal bias, i.e., that did not deviate from the straight line (*N* = 8, 2° ≤ α ≤ 18°). Equations 12 and 14 were calculated using customized FSL scripts. The influence of flip angle bias was corrected post hoc on the estimated maps of *T*_1app_ (17).

## RESULTS

### Experiment 1: Nonselective Excitation

**FIG. 2:**
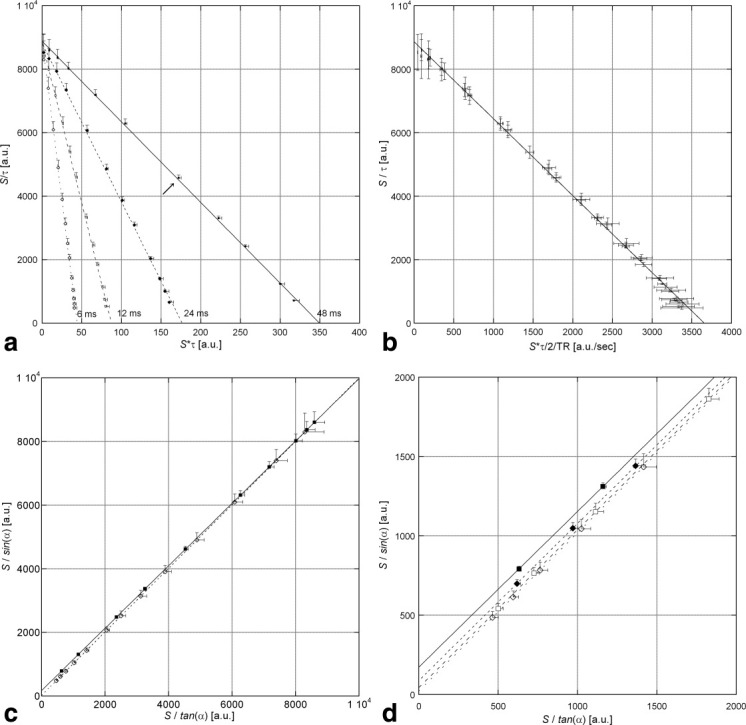
VFA measurements at different TR in 6% agar. **a**: Signals obtained at different TR are separated by the linear parameterization based on the half-angle tangent. Only positive error bars are plotted to emphasize the correlation between *x* errors and *y* errors. Close to the Ernst angle the resulting error is orthogonal to the line (arrow). **b**: VFA measurements at different TR combined into one regression over *Sτ/*2TR. **c**: The traditional linear parameterization arranges the data close to the identity. Only the data obtained at TR = 6 ms and 48 ms are shown for clarity. Data points obtained at smaller flip angles have highly correlated errors. **d**: Enlarged origin region of C to illustrate the influence of the relaxation term on the small *y*-intercepts.

The phantom data followed the linear relation between the scaled signals well (Fig. 2a). The steeper slopes were observed for shorter TR, while the amplitudes were consistent within fitting errors. The standard deviations across the region-of-interest were taken as surrogate measures of image noise. Only positive error bars are shown to illustrate the correlation of errors along abscissa and ordinate. The *T*_1_ estimates derived from ρ_1_ were between 2.40 s and 2.44 s. They were consistent within their standard errors with the value determined by inversion recovery. The standard errors of the fitted *T*_1_ decreased from 34 ms to 14 ms when increasing TR from 6 ms to 48 ms. This is in line with the TR-dependent decay of partially refocused echo signals. Since the TR's were much shorter than *T*_1_ of the agar phantom, the abscissa was scaled by 2 TR to fit *T*_1_ directly from all data points (Fig. 2b). This yielded a consistent value of 2.41 s with a small standard error of 0.01 s.

In contrast to Fig. 2a, the fitted lines at different TR could hardly be discerned when using the common linearization (Fig. 2c), because the slopes were close to one at short TR. The error bars were highly correlated. In particular for small flip angles (upper right), the error bars were almost parallel to the regression line, so the linear fit yielded a small error (about 10^−4^) for the slope, exp(–TR/*T*_1_). This yielded consistent *T*_1_ estimates, albeit with artificially low standard errors. In contrast to above, the standard errors increased with TR from 0.0002 ms to 0.0067 ms. The origin region of Fig. 2c is zoomed in Fig. 2D. The variation of the *y*-intercepts, *A*(1–exp(–TR/*T*_1_), reflects the influence of TR. Again, the errors of the fitted *A* were smaller than in Fig. 2a by more than a factor of 1000. Similar errors in *T*_1_ and *A* were observed for both methods after application of weights to account for the covariance of *x*- and *y*-errors.

### Experiment 2: Slice-Selective Excitation

**FIG. 3:**
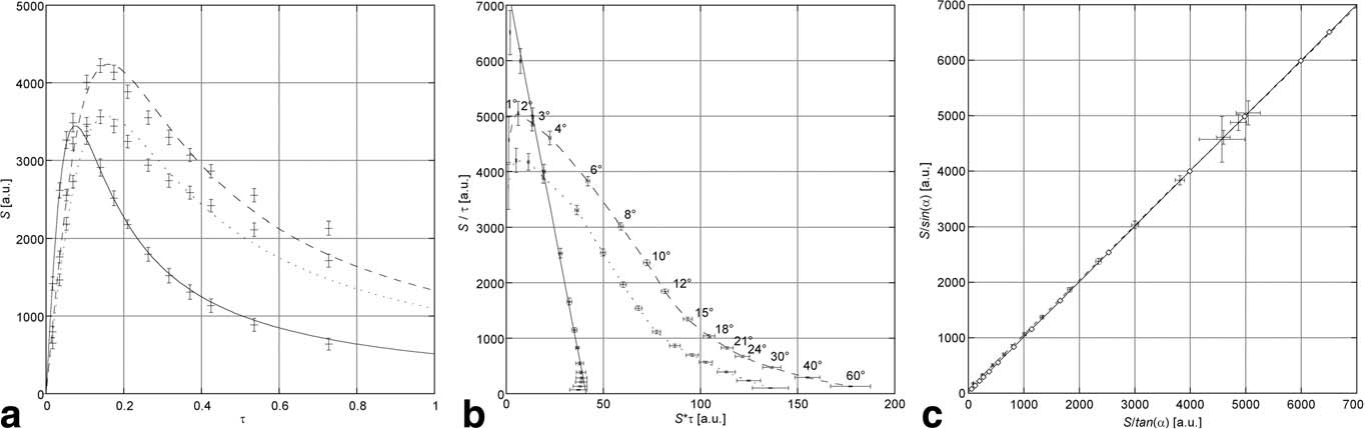
Effects of slice profile in 2D FLASH. **a**: Signal dependence of 2D FLASH on τ with fitted Ernst curves for “fast” (dashed) and “normal” (dotted) selective excitation. The signal obtained with 3D encoding (solid, “normal”) is shown for comparison to illustrate the shift of the signal maximum towards higher angles and positive residues at higher flip angle. **b**: Linear display of the data enhances the nonlinear VFA behavior of the 2D experiments. **c**: Conventional linear display of the 2D (with error bars) and 3D data (diamonds) with “normal” selective excitation. The large bias of the 2D signal is obscured, resulting in an unreasonably high linear correlation (Pearsons's *R* = 0.99999).

Slice selective VFA results in variable degrees of partial saturation across the slice profile. The signal maximum was shifted to higher flip angles when compared to 3D phase encoding (Fig. 3a). Positive signal bias was observed at higher flip angles since the margin of the profile yields a higher signal than the center. While the signal dependence on τ still remotely resembled the Ernst curve, the linear display revealed strong deviations from the 3D reference measurement (Fig. 3b). Although the “fast” RF pulse (dotted) yielded about 17% higher signal, its flip angle dependence did hardly differ from that of the “normal” RF pulse (dashed). With 3D encoding, the slice profile added to the flip angle inhomogeneity and decreased *T*_1app_ in the off-center partitions (not shown).

### Experiment 3: RF Spoiling at Varying Increment

**FIG. 4:**
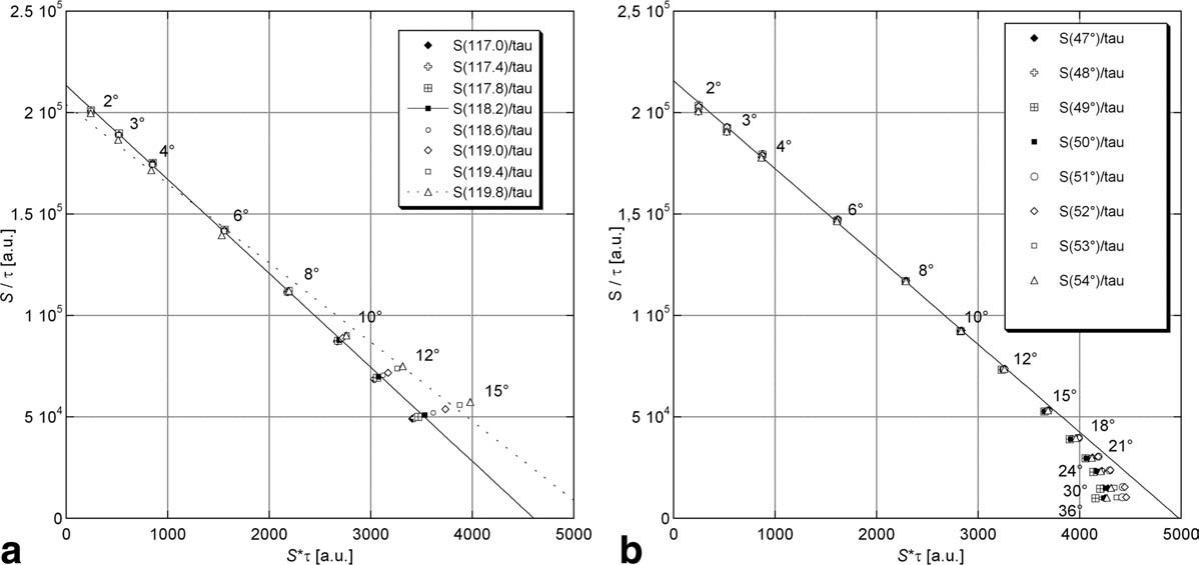
Effects of phase increment in MnCl_2_ solution. **a**: 117.0°–119.8° Deviations from the predicted line appear above 10°. **b**: 47°–54° Deviations from linear behavior (fitted to flip angles 2° to 8° at 52° phase increment) appear above 15°. Note, that signals obtained at identical flip angle fall onto lines through the origin.

Figure 4a shows the VFA measurements in MnCl_2_ solution at different RF spoiling increments. The 118.2° increment (black diamonds) conformed to a straight line (solid) as predicted by simulation (14). Already at 10°, some signals started to deviate from the linear relationship, indicating the influence of residual transverse magnetization due to partial refocusing of echo pathways. Note that the sign of residuals was not consistent for the 119.8° phase increment, leading to a widely deviating regression line (dotted). All VFA measurements at increments between 47°, and 54° followed a similar pattern (Fig. 4b). Here, linearity was well obeyed up to flip angles of 15°, followed by increasing deviations toward smaller signals.

### Experiment 4: Human Brain In Vivo

**FIG. 5:**
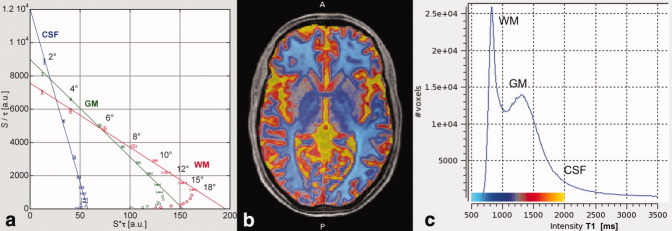
VFA measurements in vivo. **a**: Linear fit of signals from caudate nucleus (GM, squares, green), splenium (WM, diamonds; red), and lateral ventricle (cerebro-spinal fluid, diamonds, blue). Local flip angle inhomogeneities were corrected before calculating τ. Deviations from the signal equation were observed for nominal flip angles above 18° and excluded. **b**: Pseudo-color overlay of flip angle corrected *T*_1_ values to enhance the inhomogeneities within WM and deep GM. The distribution across the cortex is degraded by partial volume effects at a resolution of 1.25 mm. **c**: Whole-brain histogram of *T*_1_ values with color-scale of the overlay.

In vivo, the VFA measurement at TR = 11 ms and 50° phase increment conformed to a straight line for nominal flip angles up to 15° (Fig. 5a). Data points obtained at higher flip angle showed the negative residues typical for the 50° increment and were omitted from the linear fit. For such flip angles up to 15°, τ is practically identical with the flip angle (16) and the correction of *T*_1_ maps for the actual flip angle was performed post hoc as described in (17). RF-corrected *T*_1_ values were 0.85 ± 0.03 s for WM in the splenium, 1.29 ± 0.04 s for GM in the caudate head, and 4.58 ± 0.13 sec for cerebro-spinal fluid in the lateral ventricle. They are displayed as a pseudo-color overlay in Fig. 5b. The *T*_1_ histogram showed the expected separation of WM and GM modes (Fig. 5c).

## DISCUSSION

We derived a novel linear plot for the VFA experiment that has several advantages over the conventional method (5) that has been widely used for *T*_1_ quantification. It provides a more intuitive display of the VFA data where signal amplitude and relaxation term are represented separately as intercept and slope of the regression line. The line segment is centered on the Ernst angle, which thus can easily be identified. Since the parameterization is based on the half-angle tangent, the flip angle range up to 180° could be exploited, in contrast to just 90° with the conventional method. For flip angles exceeding 90°, however, the increasing nonlinearity of the half-angle tangent would require a correspondingly precise mapping of the flip angles. At low flip angle and TR ≪ *T*_1_, however, τ and ρ_1_ may be replaced by α and *R*_1_TR in good approximation allowing for post hoc flip angle correction (16). Under these conditions pertaining to FLASH imaging (17), the influence of TR on the slope can be removed by additional scaling of the abscissa (18). Signals obtained at different TR are combined into a single linear regression that directly yields *T*_1_. This offers additional experimental flexibility to incorporate images measured at different TR, e.g., in the context of magnetization transfer experiments (24,25,27).

Being a generalization of dual angle experiments (16,17), the linear plot yields estimates of an apparent *T*_1_ that increases with the square of the multiplicative transmit bias. Bias correction can be performed post hoc using independently acquired flip angle measurements (26–28) or by applying a unified segmentation algorithm (20) to the uncorrected maps of apparent *T*_1_ (21). Flip angle mapping constitutes an independent source of error that cannot be visually assessed by our suggested plot. The *T*_1_ values determined in the splenium and caudate of our single healthy volunteer, were above the average of a cohort study applying inversion-recovery for *T*_1_ quantification at 3T (*T*_1_ = 748 ± 64 ms in splenium; *T*_1_ = 1258 ± 55 ms in caudate; 29), but still fell into 95% confidence interval.

The greatest practical advantage of the suggested linear plot is the conspicuous identification of signal bias, i.e., deviation from the Ernst equation representing perfect spoiling. This was illustrated for the partial refocusing of echo pathways and for slice profile effects in 2D imaging (22). Signal bias is difficult to identify in the conventional plot, because this imposes a large covariance of errors, so that even strongly deviating data points are projected closer to the ideal line. This resulted in unreasonably high linear correlations which may obscure inferior data quality. Such systematic errors can be even difficult to find when fitting the nonlinear flip angle dependence, since the arbitrary signal amplitude is adjusted simultaneously. An immediate application of our method is to control the spoiling of RF quality of the FLASH sequence as implemented on one's clinical or experimental MR system as demonstrated in this study.

While the conventional linear plot may cover up signal deviation, the suggested method is highly sensitive to it and may thus be more prone to errors. In particular, incompletely spoiled echo signal became influential at flip angles lower than the theoretically derived *T*_1_-w optimum (at 2.41 τ_E_). If suitable correction techniques (14) are to be applied, one still can pursue SNR optimization. However, if such processing is not available, it is recommended to sacrifice SNR in favour of reducing systematic errors in the fitted parameter maps (as done in Exp. 4).

The contributions of spin echoes and stimulated echoes vanish in the limit of α → 0, because they depend on higher order powers of the flip angle. Since the signal bias is negative at most phase increments, it will be enhanced when approximating τ by α. Spoiling gradient areas of 280–450 mT ms / m as recommended by (15) to reach the diffusion limit can only be achieved on clinical MRI systems by increasing the spoiler duration and thus TR. Signal offsets due to Rician noise at low SNR and motion (22) will cause additional deviations. The negative residues observed at very low flip angle (1°–2°) we tentatively ascribe to additional saturation due to noise leaking into the sample from the unblanked RF power amplifier. Measurements of rapidly relaxing water moieties at ultra-short TE will also deviate from the ideal flip angle dependence (30).

A full mathematical account of the method was derived, as a generalization of the dual angle approach (16). The signal equation of partial saturation was transformed into an algebraic function of τ obtained by the half-angle tangent substitution of trigonometric functions. The relaxation term was cast into a single expression (ρ_1_) by a similar transformation, yielding a low-order rational function in τ and ρ_1_. This facilitated the use of computer algebra for deriving the regression formulae (Eqs. 12 and 14) and the propagation of image noise to the *T*_1_ and amplitude maps. As an important result we found that there was no common optimal setting for maximal SNR in both *T*_1_ and amplitude maps. Theoretical noise optimization for *N* measurements demanded a clustering of flip angles; particularly a repetition of the dual angle scheme to estimate *T*_1_ from an even *N*. This is in contradiction to results obtained with a genetic algorithm for the conventional linear parameterization (8). This may be due to behavior of the genetic algorithm or to differences in the scaling of signal noise which may affect linear regression analysis (10). Suitable weights for the residues have been derived for the suggested methods in the theory section, but as a rule these require an a priori estimate of *T*_1_. However, the effects of signal bias most often exceeded those of signal noise.

The immediate application of our method refers to the flip angle dependence of the residual signal deviations. The main rationale for acquiring signals yielding equidistantly spaced points on the linear plot—even at the cost of reduced SNR—is to define the straight line, against which signal bias at low and high flip angles can be identified and excluded from an iteration of the regression. This applies also to patient motion, which can corrupt singular volumes of the VFA series. The in vivo experiment was performed with a phase increment of 50° as implemented in the MR scanner's FLASH sequences and previously recommended for dual angle *T*_1_ mapping (14). The flip angle dependence of the signal conformed to the Ernst equation for rather small flip angles (up to 15°–20°). These may be well lower than the higher flip angle in a noise-optimized dual angle protocol. The flip angles for noise optimized *T*_1_ (Table 1) correspond to 85.3% and 14.7% of the amplitude level in *S*/τ. The latter signal may be well affected by bias, which can be corrected by suitable calibrated models (14). Alternatively, deviating data points at high flip angle data have to be identified and subsequently excluded from the analysis. The theoretical optimization does not take into account signal bias, but still confirms the intuitive insight that the precision of the amplitude benefits from acquisitions at low flip angles. High resolution amplitude maps have been shown to be useful for depicting deep brain nuclei (31). In fact, amplitude mapping corresponds to correcting the measured signals for varying relaxation effects; which is achieved here by extrapolation of *S*(τ)/τ to τ = 0. The conventional method, on the other hand, places data points closer to the *y*-axis as the flip angle approaches 90°. The contributions of partially refocused echo pathways will thus influence the estimation of *y*-intercept, and hence the amplitude maps.

Our study imposes practical consequences for the design of a VFA protocol. Upon choosing the TR and the number of measurements, data points should be evenly distributed along the regression line to fully exploit the potential of the suggested linear plot to identify bias. To account for local flip angle bias, the corresponding data points are centered on the apparent Ernst angle, that is, prior to flip angle correction. This condition cannot be met across the whole brain. The median of the apparent τ_E_ can be a suitable compromise to determine the array of flip angles from Eq. 11. This scheme may require values of fractional degrees. If *T*_1_ is to be estimated without correction other than the indispensable RF correction, it should be avoided to acquire biased signals at very low flip angles (a = 2°) and those producing partially refocused transverse coherences at higher flip angles. An iterative exclusion of such points becomes possible with our scheme.
